# Automatic detection of abnormal EEG signals using multiscale features with ensemble learning

**DOI:** 10.3389/fnhum.2022.943258

**Published:** 2022-09-20

**Authors:** Tao Wu, Xiangzeng Kong, Yunning Zhong, Lifei Chen

**Affiliations:** ^1^School of Mathematics and Statistics, Fujian Normal University, Fuzhou, China; ^2^Fujian Key Laboratory of Agricultural Information Sensoring Technology, School of Mechanical and Electrical Engineering, Fujian Agriculture and Forestry University, Fuzhou, China

**Keywords:** electroencephalography, discrete wavelet transform, multi-scale aggregation, ensemble learning, age

## Abstract

Electroencephalogram (EEG) is an economical and convenient auxiliary test to aid in the diagnosis and analysis of brain-related neurological diseases. In recent years, machine learning has shown great potential in clinical EEG abnormality detection. However, existing methods usually fail to consider the issue of feature redundancy when extracting the relevant EEG features. In addition, the importance of utilizing the patient age information in EEG detection is ignored. In this paper, a new framework is proposed for distinguishing an unknown EEG recording as either normal or abnormal by identifying different types of EEG-derived significant features. In the proposed framework, different hierarchical salient features are extracted using a time-wise multi-scale aggregation strategy, based on a selected group of statistical characteristics calculated from the optimum discrete wavelet transform coefficients. We also fuse the age information with multi-scale features for further improving discrimination. The integrated features are classified using three ensemble learning classifiers, CatBoost, LightGBM, and random forest. Experimental results show that our method with CatBoost classifier can yield superior performance vis-a-vis competing techniques, which indicates the great promise of our methodology in EEG pathology detection.

## Introduction

Electroencephalogram (EEG), which can monitor and record the electrical activity of the brain over time (Wang et al., 2017), is an advanced electrophysiological technique. EEG recordings contain an enormous amount of physiological and pathological information, which is closely associated with the well-being of the brain, making it a highly valuable tool to support doctors and other healthcare professionals in diagnosing a variety of chronic diseases. For example, it forms a basis for diagnosing stroke in the elderly (Choi et al., 2021), intending to reduce neural damage through timely intervention, or alleviating the financial burden of patients compared with other neuroimaging techniques such as computed tomography. Furthermore, a wide range of medical applications have been flourishing along with advances in technology, enabling medical professionals to utilize EEG for intelligent diagnosis of various neurological and neuropsychiatric disorders such as depression (Jiang et al., 2021), epilepsy (Subasi et al., 2017; Amin et al., 2020) and Parkinson’s disease (PD) ( Lee et al., 2022).

Typically, neurologists diagnose brain diseases or possible cerebral dysfunctions by analyzing EEG waveform in an orderly step-wise manner, and then provide a diagnostic report to the patient. The first significant step is to decide whether any abnormal patterns are present in the brain activity signals; if so, further investigation would follow suit and medical intervention may be required. Here, it is worth noting that a particular EEG can be considered as abnormal because of many reasons including, for example, the presence of obvious pathological events such as long periods of spike and wave activity, periodic lateralized epileptiform discharges (Guerrero-Mosquera et al., 2012; López et al., 2015). Currently, neurologists often follow a complex decision tree to make the discrimination (López et al., 2015). However, this process is arduous, time-consuming, and susceptible to low inter-rater agreement (Mei et al., 2017). Halford et al. (2015) found that the Cohen’s kappa with estimated reliability was 0.58 for detecting seizures in continuous EEG data, indicating moderate agreement among neurologists. The development of automated EEG analysis approaches could assist physicians in screening EEGs with the potential of replacing evaluation by human altogether in the future. They could not only reduce the physicians’ workload, but shorten the duration of consultation for each patient (Lahmiri, 2018). Thus, a reliable method for automatic clinical EEG diagnosis without human intervention is highly desirable, especially when seeking an inexpensive and remote diagnosis.

In recent years, many machine learning-based approaches have been proposed to predict and detect brain disorders using EEG signals. Most of them consist of two parts: extracting and classifying EEG features, and the former is essential for classification tasks because it can clearly influence the overall performance. In terms of feature extraction, time-frequency techniques like wavelet transform (WT) or its variants have attracted more and more attention, as on the one hand it is a more advanced and sophisticated technology that can associate spectral information with the time domain (Padfield et al., 2019). On the other hand, existing studies indicate that brain signals are non-linear and non-stationary in nature. For example, Adeli et al. (2007) pointed out that the features from the specific frequency sub-bands obtained using discrete wavelet transform (DWT) could provide better information than those from the original brain signals. Once the feature sets are determined, different types of classifiers would be designed to classify them. The commonly used classifiers include support vector machine (SVM), Riemannian geometry (RG) (Gemein et al., 2020), Light Gradient Boosting Machine (LightGBM) (Ke et al., 2017), Categorical Boosting (CatBoost) (Albaqami et al., 2021), etc. Among them, ensemble learning methods that combine multiple models for solving various classification problems are a common and popular technique and, in particular, gradient boosting-based ensemble methods have attracted great interest, due to their outstanding performance and flexibility ( Samat et al., 2022).

Currently, plenty of studies are available in the literature on the automatic diagnosis and detection of specific diseases and disorders, but few of them are concerning the diagnosis of general EEG pathology, which is considered a vital first step in conducting EEG analysis either manually or automatically (Sejdic and Falk, 2018; Albaqami et al., 2021). In particular, it can effectively decrease the false alarm rate of tasks such as seizure detection (López, 2017). Thus, we focused our investigation on the automation of this step. Despite the remarkable progress that has been achieved, some challenges remain that warrant attention and further investigation in this field. First, most existing feature-based methods only focus on feature extraction while ignoring the feature redundancy, compromising the classification accuracy and speed of machine learning methods. Second, as is documented in several recent studies (Cassani and Falk, 2020; Bodizs et al., 2021), EEG signatures are heavily influenced by the age factor. However, the vast majority of the previously published studies completely neglect the age information of patients in the classification process, which may have negatively affected their performance to a large extent.

To address the above challenges, we propose a simple yet effective method for the detection of abnormal brain signals, which can mine a compact set of features from EEG data to adequately represent the most distinguishing characteristics between target categories. Specifically, we first utilized DWT to break down the EEG signal into several wavelet coefficients and left out non-significant coefficients based on certain threshold criteria, thereby restricting the number of significant wavelet coefficients. Then, a set of features with substantial roles in improving detection performance were extracted from each selected coefficient. Finally, unlike traditional feature-based approaches, we make full use of both local and global aggregation to reduce feature dimension and redundancy. In addition, we also attempt to fuse age information with the extracted multi-scale features to further enhance the overall performance of this method. Three popular ensemble learning classifiers, i.e., RF, LightGBM, and CatBoost, are adopted to distinguish the EEGs based on the integrated features. Experiments are conducted on the widely accepted benchmark dataset, which contains EEG signals of patients with various neurological disorders. The results show that our proposed methodology compares favorably to other state-of-the-art methods in performance. In addition, we also conduct ablation studies, which demonstrate the effectiveness of our proposal. Our main contributions are summarized below:

(1) We present a novel lightweight multi-scale aggregation mechanism for precise EEG pathology detection, which extracts the discriminative multi-scale features *via* local-global hierarchical aggregation and fuses these features with patients’ age as multimodal features of brain signals. More importantly, it can significantly decrease both the dimensionality and redundancy of the features whilst improving the classification accuracy.
(2) We conduct extensive qualitative and quantitative experiments on a commonly used, standard abnormal EEG data set. The results demonstrate that our proposed approach significantly outperforms other state-of-the-art baselines. Besides, experiments also show that the age information is crucial for detecting abnormality in EEGs.

The remainder of this paper is organized as follows. In the section “Materials and methods,” we first describe the abnormal EEG dataset being used in our study, and give an overview of data preprocessing. We then discuss the DWT-based feature extraction in detail and explain how we obtain the features from EEG signals. Next, we explore how to compress the extracted statistical features to acquire significant representation. The section concludes with a discussion of different ensemble learning classifiers we utilized to facilitate classification task. In the section “Experimental results and discussions,” we discuss the extensive experiments conducted in this work and analyze the results, while conclusions and future directions are presented in the section “Conclusion.”

## Materials and methods

**FIGURE 1:**
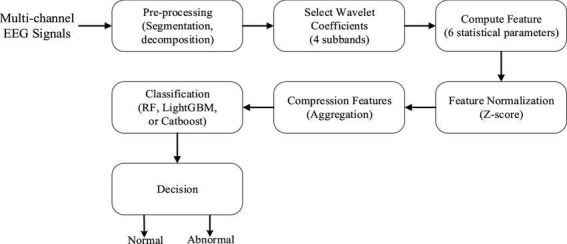
Block diagram of our proposed abnormal electroencephalogram (EEG) signal detection framework.

In this section, we propose a novel framework to automatically classify EEG recordings as either normal or abnormal. Figure 1 depicts the general workflow of our framework, which mainly comprises the following three phases. The first phase is the pre-processing which aims at ensuring data consistency. The second phase is to extract important statistical features from each optimally selected DWT coefficient and then exploit our proposed multi-scale aggregation mechanism to improve the discrimination of features. Finally, the fused features are classified using three popular ensemble learning algorithms, which can not only assess the effectiveness of the proposed technique but also choose an appropriate classifier for EEG abnormality detection. We will discuss each part in the following subsections.

### Data description and preprocessing

**FIGURE 2:**
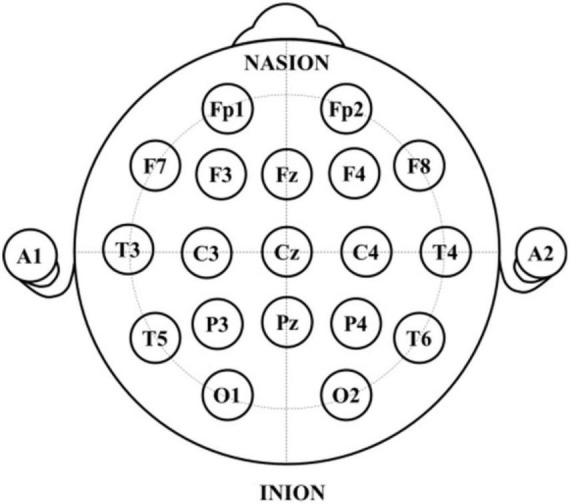
Distribution of the 21 EEG electrodes according to 10–20 system.

**FIGURE 3:**
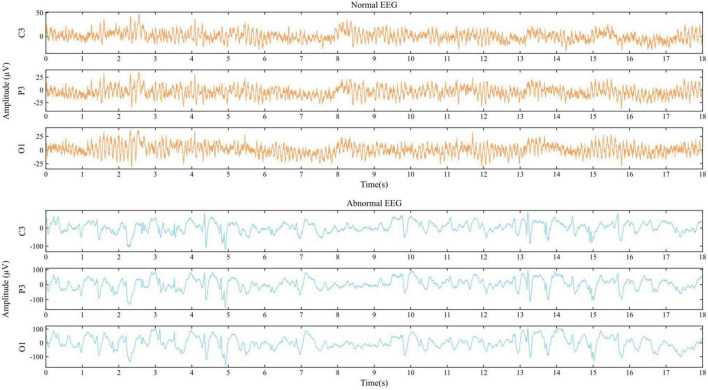
Examples of EEG signals from C3, P3, and O1.

The EEG data for this study are derived from the TUH EEG Abnormal Corpus (López, 2017), which is the publicly available dataset for the study of general EEG pathology. It is part of the TUH EEG Corpus, the largest open-source EEG dataset collected by Temple University Hospital. This corpus, as a widely used benchmark, is still being updated, and the current version is v2.0.0. To date, this dataset contains the scalp EEG data taken from 2,329 unique patients of various ages ranging from infants to the elderly. Seventy percent of reported patients are male, and the mean age of patients is around 48 years with a standard deviation of 17.89, where the maximum age was 96 and the minimum age was 7 days. The EEG data in this corpus were acquired using the common electrodes arranged in accordance with the international 10/20 position system (as shown in Figure 2) and involved at least 15 min duration of recordings for each patient, with a dominant sample frequency of 250 Hz or more. Moreover, this corpus has been segregated into two subsets, i.e., the training subset (2,717 recordings) and the testing subset (276 recordings), and meanwhile each recoding was manually labeled as either abnormal or normal by clinical neurologists, of which 1,521 were classified as normal, while the remaining 1,472 were categorized as pathological recordings, as illustrated in Table 1. Figure 3 describes typical normal and pathological EEG signals in this dataset, where the former and latter were collected from a normal 51-year-old woman and a 33-year-old male patient with refractory epilepsy, respectively. It can be seen from abnormal EEG that sharp-and-slow wave, spike- and polyspike-slow wave are present in left central, left parietal and left occipital regions, which supports the diagnosis of epilepsy to some extent. By contrast, abnormal patterns are absent from all of the channels (including C3, P3, and O1) in normal EEG. Obviously, even though neurologists can generally follow a complex decision tree to systematically evaluate the abnormality of EEG, it is a challenge to visually interpret and especially difficult to perceive signal variations (Acharya et al., 2015), due to the non-stationary and chaotic properties of this physiological signals; thus, a suitable automatic approach is highly desired.

**TABLE 1:** Number of patients and files in TUH EEG Abnormal Corpus (v2.0.0).

Description	Normal		Pathological	
		
	Files	Patients	Files	Patients
Training	1,371	1,237	1,346	893
Testing	150	148	126	105
Total	1,521	1,385	1,472	998

**FIGURE 4:**
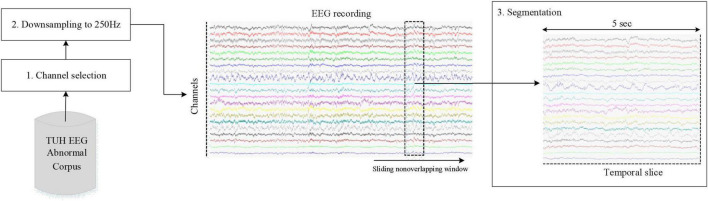
The flows of EEG preprocessing.

The EEG preprocessing included channel selection, downsampling, and signal segmentation. Specifically, the originally acquired EEG recordings are of multi-channel nature, where the minimum number of EEG channels used is 21, and 31 for the maximum. To ensure the standardization across all samples, the 21 standard electrodes considered in this paper are consistent across all recordings, as depicted in Figure 2. After which, each EEG recording is resampled with the common sampling rate of 250 Hz, aiming to make the sampling frequency of all EEG signals consistent. Finally, we segmented each channel data into 100 equal data slices of size 1250 by employing a 5-s non-overlapping sliding window and discarded the remaining EEG signals, as illustrated in Figure 4.

### Multi-resolution analysis using discrete wavelet transform

Generally speaking, EEG features can be obtained from time-domain analysis, frequency-domain analysis, or both of them. However, similar to many other biomedical signals, EEG signal is non-linear and because of its non-stationary characteristics, there may be a weak classification effect when only the frequency or time-domain characteristics are taken into account. If considering the frequency-domain features alone, while ignoring other domain characteristics, some significant information would be lost, e.g., the potential correlation between frequency content of the signal and the temporal domain. Likewise, only considering the time-domain characteristics may neglect the valuable frequency information. Nevertheless, the time-frequency analysis can effectively overcome the above disadvantages, since it is able to associate spectral information of brain signals to the time domain, thus being advantageous for EEG detection.

At present, short-time Fourier transform (STFT) and WT are two common time-frequency analysis methods. STFT, a windowed combination of Fourier transform, can transform time-domain raw EEG data into a two-dimensional time-frequency representation by utilizing a proper window that translates or slides through the whole signal, thereby making it capable of extracting time-varying spectral features from EEG signal. However, it suffers from some limitations, such as the single fixed analysis window for all frequencies which not only is difficult to determine the size in practice but also cannot make adaptive adjustments according to the time-frequency properties of brain signals. As an alternative to the STFT, the WT employs a variable sized window region when analyzing EEG signals, namely allowing signal analysis from coarse to fine multi-resolution perspective. Specifically, the WT can capture either the relevant time or frequency information by decomposing the signal into different spectral components using multi-resolution analysis. In addition, it is found to be a quite appropriate tool for capturing transient events of brain signals, such as spikes and sharp waves (Subasi et al., 2017). Up to now, the WT mainly includes continuous wavelet transform (CWT) and DWT.

Specifically, let *x*(*t*) be the signal of the *C*-channel EEG recording, the CWT is mathematically defined as follows:


$$\mathrm{CWT}\,\left(a,b\right)=\frac{1}{\sqrt{a}}\,\int_{-\infty }^{+\infty }x\,\left(t\right)\,\psi^{{}^{*}}\,\left(\frac{t-b}{a}\right)\,\mathrm{dt},$$

here, *a* and *b* are scaling and translation parameters, respectively, *CWT* (*a*, *b*) represents the wavelet coefficients, *t* is the time and ψ* represents a complex conjugation of scaled and translated versions of the continuous mother wavelet function *ψ*. The *ψ* can be compressed or stretched by the scaling parameter, and its time location can be changed using the translation parameter (Grossmann and Morlet, 1984). The high and low frequency components can be obtained by contracting or dilating the wavelet function in signal analysis, respectively; hence, it makes the CWT possible to achieve excellent time and frequency resolutions simultaneously. Practically, when continuous parameters *a* and *b* are defined as discrete values, the CWT will be referred to as DWT. This means that the wavelet’s coefficients in DWT are calculated at discrete intervals of time and scale.

**FIGURE 5:**
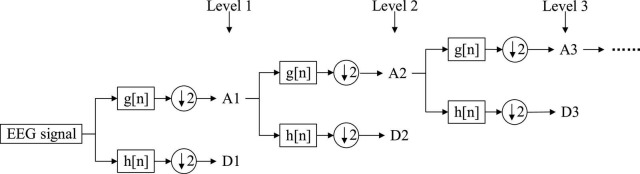
Scheme of discrete wavelet transform (DWT) decomposition.

As a special case of the CWT, the DWT decomposes the input signal into various sub-bands using filter banks which consist of complementary low- and high-pass filters, and then calculates the corresponding wavelet coefficients, where the down-sampled outputs of the low- and high pass filters correspond to the approximate and detailed coefficients, respectively. It supports a multi-level wavelet decomposition of EEG signals. Each level involves two filters with two downsamplers by 2. Figure 5 illustrates the DWT decomposition procedure, where h[n] and g[n] each denote the high- and low-pass filters, and symbols “A” and “D” represent the approximation and detail coefficients, respectively. We can observe that an original input signal is successfully divided into an approximation A1 and a detail D1, and subsequently the generated approximation is further split into a next-level approximation and detail, repeating this procedure in the lower level until the desired number of levels is reached. After *n* levels decomposition, the DWT will output (*n* + 1) coefficients, including *n* detail coefficients from D1 to D*n* and only one approximation coefficient; of these, D*n* corresponds to the frequency range of *sf*/2^*n*+1^-*sf*/2*^n^* Hz, where *sf* is the sample frequency. These coefficients offer a compact representation showing the signal’s energy distribution in time and frequency. In addition, it is worth mentioning that in the DWT, the selection of a suitable wavelet function and the decomposition levels have a significant impact on EEG analysis. There are currently a number of wavelet families available like Coiflets, Daubechies, Symlets, etc. Each of them has different members. For the other key factor, naturally, the more decomposition levels, the better frequency resolution, and the more detailed information can be yielded, with increased complexity and computation time, perhaps even feature redundancy as the trade-offs. Literature survey indicates the choice of decomposition level is determined by the signal’s primary frequency components ( Raghu et al., 2019).

### Salient feature extraction using different aggregation strategies

**FIGURE 6:**
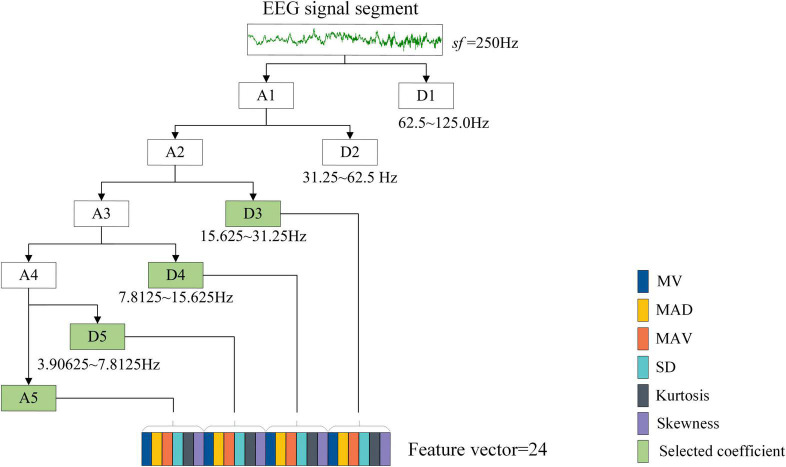
Discrete wavelet transform feature extraction from the raw EEG signals.

Initially, using DWT with Symlets wavelet, the brain signal is split into one approximation and several details. We selected the known Symlets wavelet of order 6 (sym6) in this work; as previously reported by authors (Chen et al., 2017; Frikha et al., 2021), the wavelet filter sym6 is very adequate for biomedical signal analysis because of its orthogonality nature, in particular for abnormality detection in EEGs. As for the decomposition level, it can be determined mainly based on the following consideration. Since EEG signal has multi-frequency properties, it can be commonly divided into multiple fundamental frequency bands or rhythms which are known as Delta (<4 Hz), Theta (4–8 Hz), Alpha (8–13 Hz), Beta (13–30 Hz), and Gamma (>30 Hz), respectively (Schirrmeister et al., 2017; Rahman et al., 2021); of these, the first four contain significantly discriminative information. For example, the studies of Bashivan et al. (2014) and Tsipouras (2019) indicate that the features of epileptic seizure mainly appear in the frequency range below 30 Hz. Grin-Yatsenko et al. (2010) found that theta, alpha, and beta rhythms show significant differences between depressed patients and healthy controls. Thus, it is reasonable to determine the decomposition level that makes the final obtained wavelet coefficients correlate within the valuable rhythms as much as possible. Considering the sample rate of the used EEG data is 250 Hz after preprocessing, the decomposition level was set to 5 in this work, in order to filter out irrelevant information. As more useful frequency ranges are required in EEG detection, one final approximation coefficient A5 and three details i.e., D3, D4, and D5 are chosen, as shown in Figure 6.

Although the decomposed signals can be directly concatenated into a single feature vector as the input of classifiers, these signals have been found to be particularly susceptible to noise (Soman and Jayadeva, 2015). Feature extraction is a practical and effective methodology to deal with this problem (Lee et al., 2022). By extracting a set of useful and discriminative statistical information embedded in the wavelet coefficients, it will not only achieve the purpose of characterizing EEG signals but also contribute to further reducing the dimension of feature space. Figure 6 depicts the statistical feature extraction process from EEG segment. Like the studies of Amin et al. (2020) and Al Ghayab et al. (2019), six statistical parameters used in this work are shown below:

(1) Mean of the wavelet coefficients for every sub-band (MV).
(2) Mean of the absolute deviations of the coefficients in every sub-band (MAD). This can be expressed as:

$$MAD=\frac{1}{m}\sum_{i}^{m}\left|x_{i}-u\right|,$$

where *u* represents the mean value of the wavelet coefficient with *m* data points, and *x*_*i*_ (*i* = 1, 2, …, *m*) represents the *i*-th data point in the coefficient.

(3) Standard deviation of the coefficients in every sub-band (SD).
(4) Mean of the absolute values of the coefficients in every sub-band (MAV).
(5) Skewness of the wavelet coefficients in every sub-band.
(6) Kurtosis of the wavelet coefficients in every sub-band.

The MV and MAV features are extracted for evaluating the signal frequency distribution. The SD and MAD features are measures of the variations in the frequency distribution of a signal. The skewness gives information about the degree of asymmetry in the frequency distribution and the kurtosis characterizes the sharpness of the peak of frequency-distribution curve. Overall, 6 (# statistical parameters) × 4 (#coefficients) = 24 features are generated for every single-channel EEG segment, based on which a feature vector is formed, as shown in Figure 6.

After calculating features, the next steps are normalization and to employ a new mechanism to further significantly decrease the dimensionality and increase the separability of the feature vector.

#### Feature vector normalization

**FIGURE 7:**
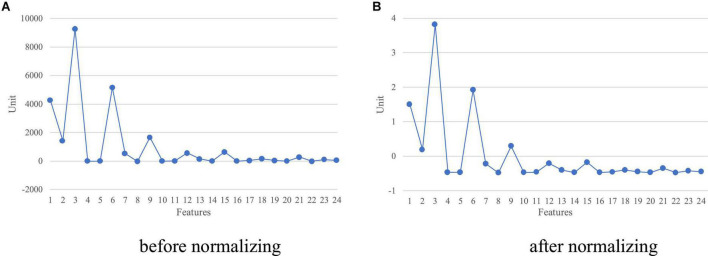
A feature vector with a size of 24 **(A,B)** before and after normalizing.

Since scalp EEG signals have arbitrary positive or negative voltage values, the statistical characteristics derived from DWT inherently have a broad range of values. Thus, the generated feature vector for each EEG sample needs to be standardized to address the problem of feature scaling and eliminate the offset effect. This can be accomplished by using Z-score normalization so that each feature vector obtained has zero mean and unit variance. Figures 7A,B depict an example of a characteristic vector before and after normalizing, correspondingly. This procedure also helps the proposed framework to reduce memory requirements and improve efficiency.

#### Multi-scale feature aggregation

After a *C*-channel scalp EEG recording being split into relatively short EEG segments using a standard sliding window, the feature computation will result in a feature matrix *M*_*i*_ ∈ ℝ^*C*×*S*×*F*^, where *i* ∈ *N* and *N* denotes the total amount of EEG recordings, *S* denotes the segment number for each EEG channel and *F* represents the total number of the extracted statistical features of each channel segment. Obviously, every EEG recording will produce a feature vector with dimension *C* × *S* × *F*, when the features derived from the selected DWT coefficients are directly flattened. In this case, if the *C* and *S* are 21 and 100 for a sample, respectively, the resultant feature vector is composed of 21*100*24 = 50,400 values. This means that the extracted features are extremely high in dimensionality and may even contain considerable redundant and unreliable information. These factors have presented a serious challenge to existing machine learning methods, e.g., performance degradation (Baig et al., 2019). To deal with these factors, dimensionality reduction is a suitable option. A small feature dimension is favorable since it produces a lower computation burden and shorter learning time.

**FIGURE 8:**
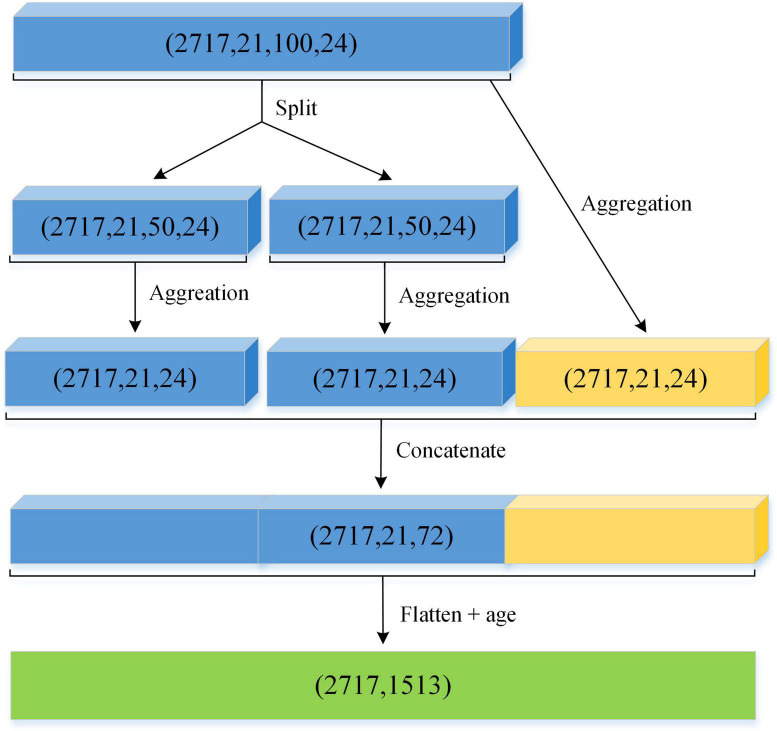
The process of the salient feature extraction for EEG pathology classification.

To further reduce the dimensionality of the constructed feature space, a rather simple mechanism is exploited, namely feature aggregation. The key idea is that a single output value computed by the aggregation function is able to summarize the information embedded in several numerical values. Considering the variabilities of the feature data over time, we adopt the standard deviation as the aggregation function because it is an indispensable tool to measure the discrete degree of the data. A large standard deviation indicates a wide distribution of the actual feature values while a small standard deviation implies the opposite. On the other hand, standard deviation aggregation has been proved to be a particularly effective method for information fusion (Corso et al., 2020). To effectively alleviate excessive feature information loss caused by the aggregation operation, this paper proposes a new multi-scale aggregation method based on global and local aggregation strategies. Specifically, for each sample, the local information takes the standard deviation of the EEG segments on the front half and rear half, respectively; the global information takes the standard deviation of all segments, where all analyzed EEG segments are ordered by time. Figure 8 illustrates a schematic representation of the proposed aggregation procedure. After feature aggregation and concatenation, the feature matrix of each EEG recording is flattened into a one-dimensional vector with dimension *C* × 3 × *F*.

In addition, a multitude of literature reveal that the spectral properties of an EEG signal are significantly associated with individual age characteristics (Namazi and Jafari, 2018; Bodizs et al., 2021). Therefore, the effect of patient age is considered in this paper. We add age as an additional feature to the flatten vector (as shown in Figure 8), and finally, obtain the feature matrix as the input of a machine learning classifier.

#### Ensemble learning classification

To build on the above premise, it is necessary to find models that can effectively process the features finally obtained, even a large amount of them. Toward this end, ensemble learning methods have become a very important technique for improving the performance of multiple existing models in the last decade. In particular, the recent three ensemble methods based on gradient boosting and its derivatives, i.e., XGBoost, LightGBM, and CatBoost, have been shown to be efficient and accurate classifiers for usage in supervised machine learning tasks. Their refinements mainly focus on both accuracy and speed. As a member of the family of gradient boosting decision trees (GBDT), CatBoost utilizes ordered boosting technique to prevent prediction shift caused by gradient bias and further improves the generalization ability. Meanwhile, different from other gradient boosting methods, CatBoost has a superior ability to handle categorical features with the lowest information loss during training time, which is often required to be completed separately at the preprocessing phase for other supervised learning methods. It is easy to deploy, fast to operate, and robust to noise. To date, CatBoost has been used and validated in many different classification tasks including EEG-based brain disorder analysis (Choi et al., 2021; Lee et al., 2022). In this work, we have compared the performance of different kinds of classifiers, and the CatBoost is the best option for this task, as shown in the latter part of this paper.

## Experimental results and discussions

In this section, we briefly summarize the experimental setup and the evaluation criteria used. Then, we go through the results that were obtained from testing the proposed scheme on the benchmark dataset, and compare the performance against other previous approaches. Finally, we elaborate on how the final results were achieved through ablation study to explain the contribution of this study. In the meantime, the effectiveness of our framework was also further examined through adjusting the number of EEG channels.

### Experimental setup

All the experiments were performed on a workstation configured with Intel(R) Core(TM) i7-8700 CPU 3.20 GHz, 64 GB RAM, and NVIDIA GeForce RTX 3090 GPU with 24 GB memory. Our scheme was implemented using the library scikit-learn (version 0.24.2) (Pedregosa et al., 2011), together with Python3 language on the Ubuntu 18.04 operating system.

As mentioned above, we used the TUH EEG Abnormal database to validate the proposed methodology. The raw EEG data were first clipped into 100 segments by sliding a 5-s window with no overlap. Then, the detail and approximation coefficients for each segment of each EEG channel were obtained by using DWT with the sym6 as the mother wavelet, where the decomposition level was 5. After that, the D3, D4, D5, and A5 coefficients were selected from the resulting coefficients (as shown in Figure 6), and meanwhile six statistical parameters (i.e., MV, MAV, SD, MAD, skewness, and kurtosis) were derived from each selected coefficient, to prepare feature vector. Subsequently, a novel multi-scale aggregation method was employed to decrease the dimension of the normalized feature vectors, and appended the age of each patient to the corresponding feature vectors after flattening. Finally, the feature vectors were forwarded to CatBoost, LightGBM, and RF classifiers. We trained classifiers on the complete training set and predicted the instances on the independent test set for model evaluation, as in most of the existing studies (Amin et al., 2019; Albaqami et al., 2021). The hyperparameters of each classifier use the default settings in all experiments, except for the parameters listed in Table 2. Additionally, this work also followed a similar experimental protocol as in Sharma et al. (2020), that is, 10-fold cross validation was employed to evaluate which classifier will perform better. Specifically, the entire dataset was randomly partitioned into ten portions, nearly equal each. Iteratively, nine portions were used for training and the remaining one was for validation. This process was repeated 10 times until all the portions were tested, and then, the average performance of all iterations was reported.

**TABLE 2:** Hyperparameters of the three models used in this work.

Model	Hyperparameters	Values
CatBoost	n_estimators	680
	max_depth	4
	learning_rate	0.0343
LightGBM	n_estimators	150
	max_depth	18
	learning_rate	0.0441
RF	n_estimators	43
	max_depth	18
	criterion	“gini”
	max_features	“auto”

Since the external class labels are available for the experimental dataset, four well-known metrics were adopted to evaluate the performance of EEG pathology detection in this article, i.e., accuracy, sensitivity, specificity and F1-score. They are currently the main metrics to describe the effect of EEG classification and are mathematically defined as follows:


$$A\,c\,c\,u\,r\,a\,c\,y=\frac{T\,P+T\,N}{T\,P+F\,P+T\,N+F\,N}$$

$$S\,e\,n\,s\,i\,t\,i\,v\,i\,t\,y=\frac{T\,P}{T\,P+F\,N}$$

$$S\,p\,e\,c\,i\,f\,i\,c\,i\,t\,y=\frac{T\,N}{T\,N+F\,P}$$

$$F\,1-s\,c\,o\,r\,e=\frac{2\,\left(T\,P\right)}{2\,\left(T\,P\right)+F\,P+F\,N}$$

where TP (true positive) is denoted by the number of EEG samples correctly identified as abnormal, TN (true negative) is denoted by the number of EEG samples correctly classified as normal, FP (false positive) is denoted by the number of EEG samples misclassified as abnormal and FN (false negative) as the samples that are wrongly labeled as normal by the classification method. Obviously, these metrics take values in the range of between 0 and 1. The larger the value, the better the classification effect. All these measures would be equal to 1, when the classification result exactly matches the raw class label.

### Comparison with previous studies

In this part, the extracted salient features were initially fed to multiple classification algorithms popular in machine learning, i.e., CatBoost, LightGBM, and RF. The purpose is to highlight the most effective classification method. Then, we compared our approach alongside several baseline and state-of-the-art approaches in literatures, including BD-Deep4 (Schirrmeister et al., 2017), AlexNet + SVM (Amin et al., 2019), the recently published methodology based on wavelet packet decomposition (WPD) (Albaqami et al., 2021). The selected competitive methods are representatives of machine learning techniques for EEG abnormality detection, and follow an analogous methodology to assess performance. We make a brief introduction about the compared approaches in the following. Sharma et al. (2020) proposed a WT-based methodology to extract three discriminatory features from each decomposed WT coefficient, and then adopted the SVM to classify them for detecting abnormality in EEGs. Their method reached a classification accuracy of 79.34% during evaluation. Gemein et al. (2020) extracted massive EEG features by using discrete Fourier transform (DFT), CWT, DWT, and connectivity between electrodes using Hilbert transform (HT), and then separately employed four classic machine learning classifiers to perform classification. The authors found that the RG produced the maximum accuracy among all classifiers. Likewise, a latest method in Albaqami et al. (2021) extracted EEG features from each selected coefficient after the WPD decomposition, and were given as input to three different GBDT models to do the same task, achieving the highest accuracy of 87.68%. In addition to the feature-based approaches, we also compared our approach against other deep learning methods, since such methods are data-driven. BD-Deep4 is an end-to-end baseline approach that uses a 4-layer convolutional neural network architecture to detect irregular EEGs, obtaining the accuracy of 84.6%. BD-TCN is an EEG-optimized deep learning network which resulted in 86.2% accuracy. Amin et al. (2019) employed the popular pre-trained model AlexNet to detect abnormality in EEGs. After transfer learning and fine-tuning, the final output layer in the model was replaced by SVM, achieving a classification accuracy of 87.32%.

**FIGURE 9:**
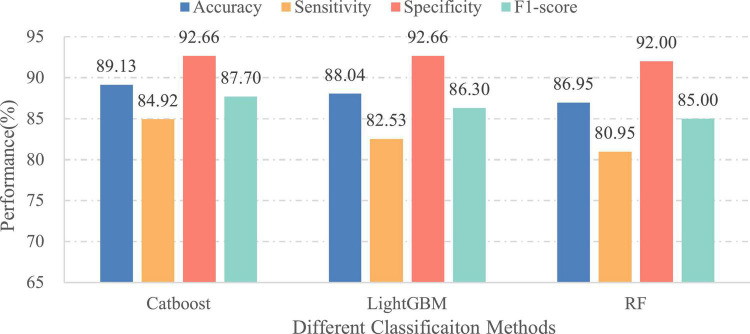
Performances of different classifiers over independent final testing set.

In the first experiment, our model with the CatBoost, LightGBM, and RF classifiers achieved cross-validation accuracies of 81.39, 80.78, and 79.38%, and cross-validation F1-scores of 80.55, 79.72, and 78.18% on a widely used real-life EEG dataset, respectively. From these results, we find that the first two classifiers clearly outperform the best performance of the baseline method (78.3% accuracy and 79.53% F1-score) (Sharma et al., 2020). In the second experiment, we trained classifiers on the complete training set, and then evaluated them on the held back test set. The obtained results with each classifier are given in Figure 9. Overall, our proposed salient feature extraction technique can make the classifiers significantly better than the feature-based and deep learning baseline methods in multiple evaluation metrics. We also observe that the CatBoost classifier obtained the highest performance with 89.13% accuracy among the three ensemble approaches. The second best is the LightGBM which exhibited approximately the same performance in accuracy. Interestingly, both methods also performed better than our previous work, which employed a deep learning algorithm with one-dimensional convolutional neural networks to detect anomalous events in EEGs (Wu et al., 2021). By contrast, the major benefit of our new approach is that multi-scale salient features are extracted from the raw brain signals before performing classification. Combining with the cross-validation results, we can clearly conclude that the suggested method based on multi-scale features and CatBoost classifier is a viable method for EEG pathology detection. More details about its classification performance are depicted in Table 3. The outcome led us to determine whether our methodology is indeed suitable for further analysis of EEG data. In addition, we can observe from the outcomes of each classifier that there are distinct differences with respect to the sensitivity and specificity indexes. The reason is that the amount of samples per category in training set is unbalanced (see Table 2). This effect has been demonstrated in previous studies as well (Gemein et al., 2020; Albaqami et al., 2021).

**TABLE 3:** Confusion matrix for the proposed CatBoost-based method.

		Predicted label			
		
		10-fold cross validation		The default test set	
			
		Normal EEG	Pathological EEG	Normal EEG	Pathological EEG
Actual label	Normal EEG	1282 (TN)	239 (FP)	139 (TN)	11 (FP)
	Pathological EEG	318 (FN)	1154 (TP)	19 (FN)	107 (TP)

Next, we compared our methodology against the existing studies using the same EEG data set, as listed in Table 4. It can be seen that our CatBoost-based methodology is obviously superior to the other seven approaches. First, the classification accuracy of our methodology is 9.78% higher than that of the feature-based baseline approach and 4.53% higher than that of the initial deep learning approach, showing a consistent improvement compared with both of them. Second, the overall performance of the proposed methodology also outperforms the existing advanced approaches based on handcrafted feature. Especially combining the results obtained in previous experiments, we find that the classification accuracy of our method increases by 1.45% compared to the latest approach under the same classifiers, which further demonstrates our methodology’s effectiveness and efficiency in overcoming the weakness of conventional EEG pathology detection methods. Albaqami et al. (2021) used WPD to break down the EEG signal into 8-level wavelet decomposition and then extracted a large number of statistical features from 16 selected components; however, it is well-known that WPD is a generalization of the DWT and has higher time complexity. In contrast, our method requires fewer decomposition levels and chooses much fewer coefficients according to domain knowledge, which greatly decreases the number of statistical characteristics extracted. The increasing dimension of the features usually not only produces some redundant or irrelevant characteristics which make feature reduction more difficult, but also can decrease the classification accuracy. Thus, the results in Table 4 suggest that the extracted features in this work are appropriate for abnormality detection in EEGs.

**TABLE 4:** Performance comparison of our proposed approach with previous approaches.

References	Accuracy (%)	Sensitivity (%)	Specificity (%)	F1-score (%)	Architecture	Features
Sharma et al., 2020	79.35	69.84	87.33	75.53	SVM	WD
Gemein et al., 2020	85.86	77.77	92.66	83.40	RG	DFT CWT DWT HT
Albaqami et al., 2021	87.68	83.33	91.33	86.06	CatBoost	WPD
Albaqami et al., 2021	86.59	81.74	90.66	84.77	LightGBM	WPD
Schirrmeister et al., 2017	84.6	75.9	91.9	82.0	BD-Deep4	
Amin et al., 2019	87.31	78.57	**94.67**	84.97	AlexNet + SVM	
Gemein et al., 2020	86.2	79.7	91.6	83.6	BD-TCN	
This study	**89.13**	**84.92**	92.66	**87.70**	CatBoost	DWT

The best results are highlighted in bold.

Also, comparing the results of our methodologies with the two latest CNN-based approaches (Amin et al., 2019; Gemein et al., 2020), it can be observed that our approaches with the CatBoost classifier and the LightGBM classifier achieved better classification performance. In particular, our CatBoost-based approach improves the classification accuracy rate by over 1.8% and F1-score by 2.73 compared to the transfer learning-based approach which used massive amount of additional unpublished EEG data during training. More interestingly, even though our method with the RF classifier is less accurate than the latest deep learning approaches, its F1-score and sensitivity are higher than both of them. These results demonstrate that our proposed feature extraction technique can effectively generate the highly discriminative features which contribute to abnormality detection. In addition, for the deep learning method, the complexity of the network structure, the number of learning parameters and the duration of the model training process will increase at the same time. So, it is worth mentioning that compared with existing deep learning approaches, our methodology requires much less parameters to be tuned.

Moreover, it can be seen from the analysis of results of the experiment that compared to the benchmark methods, the proposed methodology achieves a better sensitivity while keeping the comparative F1-score. To our knowledge, the high sensitivity, which can ensure the precise identification of abnormal EEGs, is crucial in the field of medicine, especially for automatic medical screening methods (Golmohammadi et al., 2019; Gemein et al., 2020). Therefore, the above result suggests that the proposed feature extraction may not only be highly optimal but also plays an important role in constructing a more compact and less redundant feature space which enables the classifier to achieve better prediction performance.

In summary, the proposed methodology provides a satisfying evaluation result in F1-score and clearly outperforms the other state-of-the-art approaches. Meanwhile, our results are also consistent with Gemein et al.’s (2020) findings that EEG pathology detection accuracies are between 81 and 86%, just like the case of RF; however, our present work increases the limit by more than 3%. And beyond that, our work is obviously different from existing studies in literature. For example, Al Ghayab et al. (2019) extracted 10 feature types including first quartile, second quartile and range to differentiate the epileptic EEG signals. Similarly, Gemein et al. (2020) extracted 50 feature types in six domains for detecting abnormality in EEGs. In addition, we can observe from Table 4 that even though deep learning networks have an impressive performance in other fields (e.g., computer vision) and are expected to improve the capability of EEG abnormality detection, many studies still use traditional feature engineering techniques. To our knowledge, methods based on the features and conventional classification algorithms, such as CatBoost, are more appropriate to deal with the scarcity of data, and yet deep learning methods generally require large volumes of labeled training data to ensure their normal capacity for discrimination, as well as the generalization power and robustness of the models. This is one of the main reasons why we adopt the feature-based technique to solve the binary classification problem of EEGs in this work.

### Ablation study

The core idea of our methodology lies in integrating salient EEG features and patient age. In this subsection, we provide three additional experiments in the real-life EEG abnormal dataset for in-depth analysis. The first experiment is to verify the importance of feature redundancy reduction by testing different feature aggregation schemes. The second experiment is to investigate the effect of patient age on EEG pathology detection by performing our proposed approach with and without age. The third experiment is to further sufficiently explore the efficacy of the proposed technique, through adopting a region-reduction based experimental strategy.

#### Effect of feature redundancy reduction on performance

To evaluate the influence of feature redundancy reduction on EEG abnormality detection, we conducted ablation experiments by removing each part of the proposed multi-scale aggregation mechanism. Here, four different cases were considered and compared. These cases are as follows:

Case 1: without feature aggregation process, which means that the statistical features derived from DWT analysis and age of patients are directly concatenated into a characteristic vector and then fed to an ensemble learning classifier.
Case 2: ignoring the local feature aggregation information, which means that the aggregation process only considers the global aggregation and age information.
Case 3: ignoring the global feature aggregation information in our aggregation process.
Case 4: with the global, local and age information considered in the aggregation process as shown in Figure 8.

In each case, the extracted statistical features are the same before performing the aggregation operation, and the classifier used is CatBoost. To better understand the influence of feature redundancy, the execution time for training and testing taken by this experiment are also provided. Table 5 lists the experimental results of each case. The best outcomes for each column of this table are highlighted in bold. The reported training time mainly includes the time needed for feature aggregation and classifier training, whereas the testing time involves the calculation of the aggregation features as well as the classification itself.

**TABLE 5:** Performance comparison for the four different cases.

	Accuracy (%)	Sensitivity (%)	Specificity (%)	F1-score (%)	Training time (s)	Testing time (s)
Case 1	81.15	75.39	86.00	78.51	261.588	2.494
Case 2	86.59	83.33	89.33	85.02	**9.808**	**0.061**
Case 3	84.78	79.36	89.33	82.64	12.553	0.091
Case 4	**89.13**	**84.92**	**92.66**	**87.70**	15.283	0.154

The best results are highlighted in bold.

As Table 5 shows, the cases adopting different scale aggregation achieved significantly higher accuracy rate, which indicates the importance of feature redundancy reduction to improve classification performance. Especially, the Case 4 improved the accuracy by 2.54 and 4.35% compared to Case 2 and Case 3. Even though Case 4 took slightly more execution time, it yielded a higher classification effect, which is worthwhile. In addition, we can notice that Case 1 attained a relatively poor classification accuracy with high training and testing time. This is because the wavelet-based features derived from multichannel EEG signals are of high dimensionality or large size and meanwhile contain redundant or unrelated information, which is not conducive to the subsequent classification processing. Nevertheless, we also observe that Case 1 has an approximately 2% higher classification accuracy than the baseline method based on hand-crafted features (Sharma et al., 2020). It suggests that the DWT-based feature extraction technique is effective when applied to analyze EEG signals.

In a nutshell, the experimental results demonstrate that our proposed multi-scale aggregation mechanism can significantly reduce feature dimension and redundancy without losing important information. In addition, the results also support the fact that not all characteristics are relevant or beneficial, and removing these usually contributes to enhancing the performance of machine learning classifiers whilst greatly reducing the computational burden.

#### Importance of age information

**FIGURE 10:**
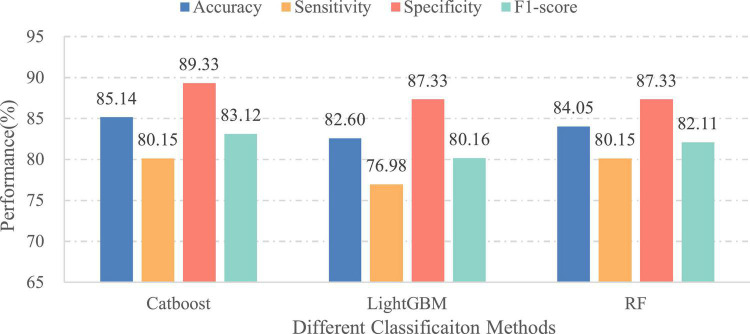
Performances of different classifiers over the independent final testing set (without age information).

In this part, we verified the importance of patients’ age for EEG pathology detection. Therefore, two different cases have been considered: (1) the final feature vectors obtained in the aggregation process include the age of patients and (2) the patients’ age is excluded from the feature set before performing classification. Meanwhile, in order to eliminate the influence of the classifier itself on the comparison of classification results, different ensemble learning algorithms are employed under the same set of features. For this purpose, the classifiers used in this experiment are the same as in the initial experiment. Figures 9, 10 illustrate the classification results of three different approaches for the first and second cases, separately. As seen from the figures, the classification effect for each classifier has greatly improved by considering patient age in EEG pathology detection task, e.g., for the CatBoost classifier, the obtained classification accuracy was 85.14% along with 80.15% in sensitivity and 83.12% in F1-score in the second case, while by adding age, the performance increased by 3.99, 4.77, and 4.58%, in accuracy, sensitivity and F1-score, respectively. Similar comparison results can also be observed for another two classifiers. This shows that considering age in this way is able to yield a statistically significant performance gain for EEG abnormality detection. Such a result is consistent with a recently reported study, where the researchers attempted to improve the accuracy of resting state EEG-based Alzheimer’s disease (AD) diagnosis by adding the age of patients in the classification process (Cassani and Falk, 2020). They found that using age as an additional feature can contribute toward the classification of healthy normal elderly controls vs. mild-AD patients. Thus, from the overall results, we can conclude that the age information is useful for EEG analysis.

#### Performance comparison under different electroencephalogram channels

To further verify the validity of our proposed methodology for abnormal EEG detection, a commonly used region-reduction based experimental approach is adopted in this work, similar to the studies of Ghosh et al. (2021) and Liu et al. (2021). Specifically, the EEG channels selected were first divided into six different regional areas, which consisted of frontal (FP1, FP2, F7, F8, F3, F4, FZ), temporal (T3, T5, T4, T6), ear (A1, A2), parietal (P3, P4, PZ), occipital (O1, O2), and motor cortex (C3, C4, CZ). We then assessed the classification performance of the proposed approach based on its ability to classify the features extracted from the EEG channels covering part of or all brain regions. Note that the age of patients was added to the final feature vectors at every evaluation. Moreover, in order to provide a thorough and more reliable analysis, the classification process was carried out using two classifiers that exhibited better performance in the initial experiment. Table 6 illustrates the experimental results of both ensemble learning models.

**TABLE 6:** Validating the effect of EEG electrodes on the proposed method performance.

Brain regions	CatBoost		LightLGB	
		
	Accuracy (%)	F1-score (%)	Accuracy (%)	F1-score (%)
Frontal + Temporal + Occipital + Parietal + Ear + Motor Cortex	89.13	87.70	88.04	86.30
Temporal + Occipital + Parietal + Ear + Motor Cortex	83.69	81.78	82.60	80.16
Frontal + Occipital + Parietal + Ear + Motor Cortex	85.86	83.54	86.59	84.77
Frontal + Temporal + Parietal + Ear + Motor Cortex	88.40	86.88	86.95	84.87
Frontal + Temporal + Occipital + Ear + Motor Cortex	86.95	85.12	87.68	85.95
Frontal + Temporal + Occipital + Parietal + Motor Cortex	86.59	84.64	85.86	83.81
Frontal + Temporal + Occipital + Parietal + Ear	85.14	83.12	85.86	83.95

From the Table 6, we can see that despite the lack of data on partial EEG channels, our proposed framework can still classify EEGs well, and in most cases achieve better results than the baseline method based on deep learning. In particular, when discarding the EEG signals from the occipital region, our proposed method using the CatBoost classifier still yields better classification accuracy than the recently proposed methods (Albaqami et al., 2021). These results further provide a stronger indication that our framework is effective in EEG pathology detection task, and has the ability to extract highly compact and discriminative features from the raw EEG signals. In addition, the results of experimental analysis also suggest that with the same classifier, different combinations of EEG channels could bring different classification accuracies. This implies that, for EEG pathology detection, various brain regions have different effects on the results; of these, the frontal region seems to be more significant in comparison with other regions, since excluding EEG signals from this area will result in the maximum performance loss, e.g., LightLGB F1-score decreased by 6.14% (from 86.30 to 80.16%) by ignoring corresponding signals. Interestingly, this is in alignment with previous research findings in other domains (Li et al., 2008; Zangeneh Soroush et al., 2020), e.g., Liu et al. (2021) found that among brain areas, the frontal area is the most influential for EEG-based driving fatigue detection. Although we did not exhaust all the combinations of brain regions due to the multitude of possibilities, the current results suggest that some specific areas, including the frontal and motor cortex, play a crucial role in EEG pathology decoding.

## Conclusion

In this paper, we presented a novel automatic detection framework based on multi-scale features and ensemble learning to solve the binary classification problem of EEGs. Different from most existing feature-based methodologies, our approach adopts a lightweight multi-scale aggregation mechanism to not only greatly reduce the feature redundancy but also minimize the computational complexity for post-processing, which is the core innovation of our framework. In addition, the age information of patients is combined with the extracted multi-scale features for further enhancing the discrimination of characteristics. The fused characteristics are classified using three different popular classifiers. Extensive experimental results show that the proposed framework yields superior performance vis-a-vis competing techniques on the same dataset, which firmly demonstrates the validity and feasibility of the proposed technique. Moreover, our experiments also indicate that the patient age is crucial for differentiation of normal versus abnormal EEGs, and therefore the age should be taken into consideration, be it by the feature development, or in the classification process.

There are many directions that are clearly of interest for future exploration. One avenue is to extend the current methodology to multiple classification scenario, that is, classifying multiple brain-related disorders based on salient feature extraction and ensemble learning. Our further efforts will also include combining other demographic and physiological factors (e.g., gender, blood pressure, etc.) together with the EEG signals to enhance the discrimination ability.

## Data availability statement

Publicly available datasets were analyzed in this study. This data can be found here: https://isip.piconepress.com/projects/tuh_eeg/html/downloads.shtml.

## Author contributions

TW, XK, YZ, and LC conceptualized the study and reviewed and edited the writing. XK and LC contributed to the funding acquisition, the supervision, and the validation of the study. TW developed the software and carried out the experiment. All authors contributed to the article and approved the submitted version.
